# Fusobacterium Necrophorum Septicemia Secondary to an Ovarian Abscess: A Case Report

**DOI:** 10.7759/cureus.26047

**Published:** 2022-06-17

**Authors:** Amanda Morrall, Uwe Schmidt

**Affiliations:** 1 Internal Medicine, OhioHealth Doctors Hospital, Columbus, USA; 2 Infectious Disease, Freeman Health Systems, Joplin, USA

**Keywords:** lemierre's syndrome, necrobacillosis, ovarian abscess, fusobacterium necrophorum, case report

## Abstract

*Fusobacterium necrophorum *is part of the normal oropharyngeal flora and can result in a life-threatening systemic infection known as Lemierre's syndrome. A rare presentation of *F. necrophorum *infection is seen in the female genital tract and is typically due to obstetric infections. Here we present a unique case of *F. necrophorum *without traditional features of Lemierre's syndrome with the female genital tract as a primary site.

A 50-year-old female presents with a two-month history of nausea, vomiting, abdominal pain, and weight loss. She ultimately developed bilateral lower extremity necrotizing fasciitis, colonic perforation, and a left chest wall abscess. Blood and wound cultures were found to be positive for *Fusobacterium necrophorum*. Imaging revealed a left ovarian mass along with a left upper lobe nodule. She had no history of oropharyngeal infections or symptoms. Imaging was also negative for deep neck space abscesses or thrombophlebitis. The patient was treated with ceftriaxone and metronidazole and clinically improved. In conclusion, *F. necrophorum *is a potentially life threatening infection and should be considered when dealing with ovarian abscesses or masses.

## Introduction

*Fusobacterium necrophorum* is a Gram-negative, anaerobic rod. It is part of the human gastrointestinal tract and is often associated with head and neck infections, localized abscesses and a potentially life-threatening disease called Lemierre's syndrome [1,2]. Lemierre's syndrome starts as an oropharyngeal infection, followed by septic thrombophlebitis of the internal jugular vein resulting in septicemia and septic emboli [1]. Few* F. necrophorum *cases are found in the female genital tract and those that do are most commonly associated with obstetric procedures [3]. Here we report a case of an otherwise healthy 50-year-old female who developed *F. necrophorum* septicemia originating from the genital tract without recent birth or obstetric procedures.

## Case presentation

**Figure 1:**
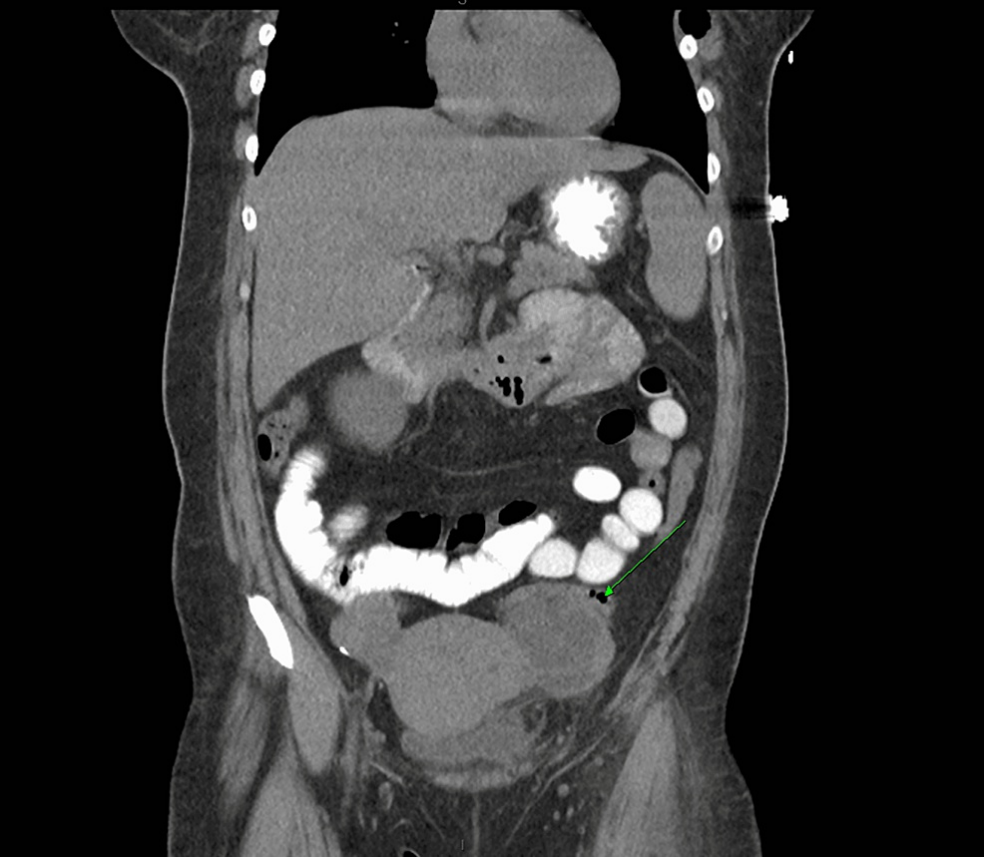
Computed tomography without contrast of the abdomen in coronal view. The green arrow indicates left ovarian mass.

**Figure 2:**
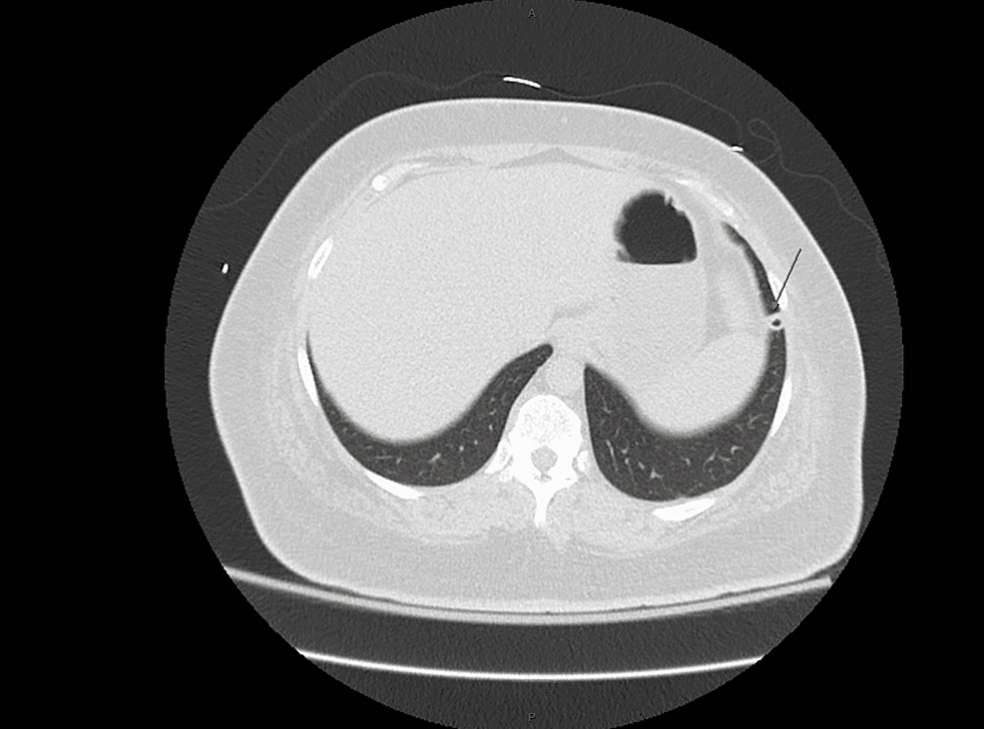
Computed tomography without contrast of the chest in transverse view. Black arrow indicates left pulmonary nodule.

**Figure 3:**
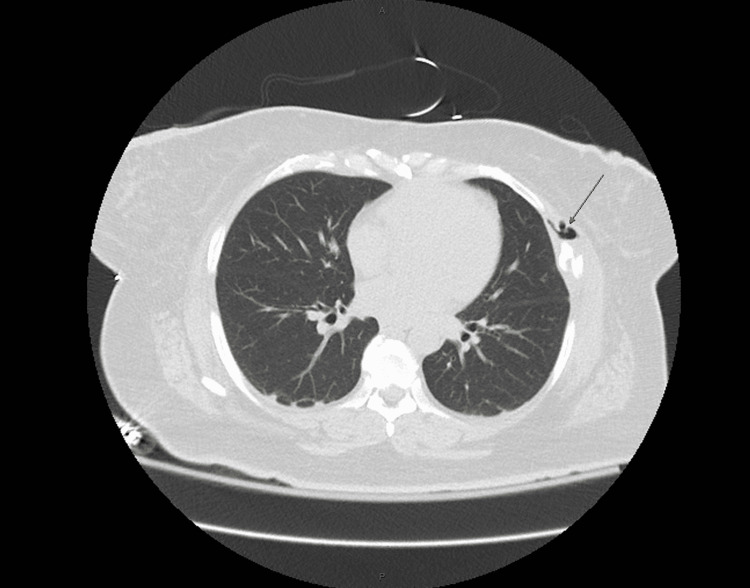
Computed tomography without contrast of the chest in transverse view. Black arrow indicates left anterior chest wall abscess.

A 50-year-old female with a past medical history significant for hyperlipidemia and obesity presented to the emergency department with two months duration of nausea, vomiting, left lower quadrant progressive abdominal pain, and diarrhea. A review of systems was notable for fevers, chills, night sweats, and an unintentional 70-pound weight loss. Vital signs on admission were significant for a fever of 101.6°F. Labs were significant for a hemoglobin of 13.2, platelets of 104,000, and white blood cell count of 6.9 with 86% neutrophils. Her BUN was 67, creatinine was 3.6, aspartate aminotransferase was 245, alanine transaminase was 59, and glucose was 106. Lactic acid levels were elevated at 5.6 and procalcitonin was 186.26 (Table 1). CT abdomen and pelvis revealed a 6.2 cm x 4.8 cm x 5 cm mixed solid and cystic mass in the left ovary/adnexa (Figure 1), a 6 mm x 6 mm pulmonary nodule in the left lower lobe with central lucency (Figure 2), a 6 cm x 2.5 cm soft tissue mass with bony destruction of the left anterior chest wall (Figure 3), and retroperitoneal lymphadenopathy. She developed hypotension refractory to fluid resuscitation, so she was admitted to critical care and started on vancomycin and meropenem empirically. 

**Table 1:** Patient's lab values upon admission to hospital.

	Patient's Lab Values	Normal Lab Value
Hemoglobin	13.2 g/dL	12-16 g/dL
Platelets	104 x 10³/ µL	150-400 x 10³/ µL
White Blood Cell	6.9 x 10³/µL	4.5-11 x 10³/ µL
Neutrophil (%)	86%	54-62%
BUN	67 mg/dL	7-18 mg/dL
Creatinine	3.6 mg/dL	0.6-1.2 mg/dL
AST	245 IU/L	9-32 IU/L
ALT	59 IU/L	19-25 IU/L
Glucose	106 mg/dL	70-110 mg/dL
Lactic Acid	5.6 mmol/L	0.5-1 mmol/L
Procalcitonin	186.26 ng/mL	0.10-0.49 ng/mL

The following day, she began to experience worsening abdominal pain and bilateral leg pain. She developed erythema, mottling, ecchymosis, and edema in her posterior right thigh and anterior left thigh. A CT of her bilateral lower extremities was obtained which showed gas in the soft tissue and fascia surrounding the muscles of her right posterior leg and left quadricep. She was urgently taken to the operating room for bilateral lower extremity debridement for necrotizing fasciitis. She was also started on clindamycin. Blood cultures and wound cultures of the bilateral thighs returned positive for *Fusobacterium necrophorum*. Antibiotics were transitioned to ceftriaxone and metronidazole. 

A CT scan of the soft tissue of the neck was negative for abscess, internal jugular vein thrombosis, mass, or lymphadenopathy. On day 13 of hospitalization, she reported a significant increase in abdominal pain and vomiting. Her physical exam was significant for diffuse tenderness with rebound and absent bowel sounds. A repeat CT abdomen and pelvis was obtained which showed severe pneumoperitoneum. She was emergently taken to the operating room for an exploratory laparotomy with left salpingectomy and oophorectomy, and a sigmoid colectomy with colostomy for a colon perforation in the vicinity of the left ovary. Pathology of the left ovary was negative for malignancy and consistent with an abscess. 

She returned to the operating room two days later for abdominal wall closure. She also underwent additional surgery for closure of her bilateral leg wounds. The patient experienced persistent nausea and vomiting during hospitalization. She was switched to ertapenem as it was believed the metronidazole was the cause of nausea and vomiting. She continued to improve and was ultimately discharged to a rehabilitation facility on a six-week course of ceftriaxone for osteomyelitis of the left rib.

## Discussion

The *Fusobacterium* genus is an anaerobic, non-spore-forming, Gram-negative bacillus commonly found in the oral cavity but also in the female genital tract [4-6]. Of this genus, *F. necrophorum *is considered the most virulent [4]. *F. necrophorum *has been divided into two subspecies: *F. necrophorum *subspecies *funduliforme *and *F. necrophorum *subspecies necrophorum [4]. The necrophorum subspecies are associated with human infection in comparison to the funduliforme subspecies which are more common in other mammals [4].

Human infections commonly present as pharyngitis, tonsillitis, cervical adenopathy, or localized abscesses [1,6]. It can also present systemically as a potentially life-threatening condition called Lemierre's syndrome [5,6]. Lemierre's syndrome was described in 1936 by Dr. Lemierre as a tonsillar abscess in an otherwise healthy young adult causing thrombophlebitis which ultimately spreads to the internal jugular vein resulting in septicemia [2]. The most common metastatic presentation is pulmonary involvement followed by joint involvement [1]. Other less common sites are muscle, liver, skin, spleen, and cardiac involvement [1]. 

To diagnose invasive *F. necrophorum*, anaerobic blood cultures should be obtained. Gram staining will most likely show a short coccobacillus with an occasional long filamentous form [7]. However, cultures of *Fusobacterium* can take up to five to eight days to grow [8]. Treatment for *F. necrophorum* is typically prolonged. *F. necrophorum *responds well to anaerobic antibiotics such as metronidazole, amoxicillin-clavulanate, clindamycin, or cefoxitin [7]. Intravenous antibiotics are recommended until the patient's fever subsides or neck swelling resolves with up to an additional four weeks of oral therapy [7].

The mortality rate from *F. necrophorum* infections can be high [1]. With pulmonary involvement being the most common metastatic presentation, it is important to obtain repeat chest CTs for early detection and drainage of severe complications such as empyema and effusions [7]. Another important consideration is surgical drainage of abscesses or fluid collections. Up to 25% of tubo-ovarian abscesses may need surgical intervention and drainage [4]. With prompt surgical care, emergencies such as ruptured abscesses can be prevented. 

To the best of our knowledge, this patient presentation is an exceedingly rare presentation of *F. necrophorum* septicemia. Gynecological infections due to *F. necrophorum* infection are exceedingly rare and are most often associated with obstetric procedures leading to tubo-ovarian abscess or uterine abscess [4,7]. In our review of case reports, there is a report of salpingitis, left internal iliac vein thrombosis, septic pulmonary emboli, and *F. necrophorum* bacteremia after first sexual intercourse [9]. Also, there is a report of a 20-year-old virgin who had a left ovarian abscess secondary to *F. necrophorum* which was preceded by a sore throat, neck pain, and fever, consistent with Lemierre's syndrome [8].

Many define Lemierre's syndrome as an infection arising from the oropharynx. However, there remains debate regarding if proven thrombophlebitis of the internal jugular vein is required or if septic pulmonary emboli or lung infarctions are an acceptable substitute [1]. With regards to our patient, she had no evidence of oropharyngeal symptoms or internal jugular vein thrombosis but did have pulmonary septic emboli. Her left ovarian abscess seems to have been the initial infection, resulting in septicemia, pulmonary septic emboli, chest wall abscess with rib osteomyelitis, and necrotizing fasciitis of both thighs. Ultimately, it remains speculative if the ovarian abscess started as a silent Lemierre's syndrome or as a gynecological infection. In conclusion, this case demonstrates the virulence of *F. necrophorum* combined with multiple serious metastatic infections.

## Conclusions

*F. necrophorum* is most commonly associated with oral infections but it is imperative to keep it in the differential for ovarian abscesses/masses. Lemierre's syndrome is quite often overlooked and can be challenging to diagnose in the absence of classic findings such as thrombosis of the internal jugular vein. However, it is a severe and potentially life-threatening infection if allowed to spread throughout the body. With prompt recognition and diagnosis, complications such as lung abscesses, septic arthritis, pyomyositis, subcutaneous abscesses, liver abscesses, peritonitis, and endocarditis can be avoided.
